# Supplementary material for: Self-care practices and health-seeking behaviours in patients with dengue fever: A qualitative study from patients’ and physicians’ perspectives
Source: PLoS Negl Trop Dis. 2023 Apr 27;17(4):e0011302. doi: 10.1371/journal.pntd.0011302 (PMC10168551; doi:10.1371/journal.pntd.0011302)
Supplement: S1 Interview — (DOCX) [file pntd.0011302.s001.docx]

**Topic guide for doctors**

**Study title:** Exploring self-care practices and health-seeking behaviours of patients with dengue fever: A qualitative study from the perspectives of patients and doctors

Preamble:

• Ice-breaking session

• Explain purpose of session

• Interested in personal views and opinions

• No right or wrong answers

• Permission to refuse answer questions

• Views will be kept confidential

• Explain the necessity to audiotape (to help analyse your views in detail later on)

• Get demographic data and take electronic consent (for both interview and audiotaping)

• Any questions before we start?

1. Please describe your previous experience when seeing dengue patients.

-how do you decide whether to treat inpatient/outpatient?

-do you use any guidelines?

-what is the advice given to patients if you are seeing them outpatient?

-did any patient ask you about their home remedies/traditional way of treating dengue fever? What is your opinion towards the complementary and alternative medicine circulating in the community?

-how do you advise your patient about it? (do you think patients retain the information?)

1. Please describe the challenges that you faced when treating dengue patients.
2. What is your opinion on the compliance of dengue outpatients to their daily follow up?
3. Do you have any experience in handling difficult dengue cases?

-Describe why is it difficult

-related to patient? Healthcare system?

1. How do you perceive the susceptibility of severe dengue among dengue patients that you have seen?

-opinions on awareness of severe dengue among patients

-patients’ knowledge about severe dengue and its seriousness

-do you think the mortality of dengue cases are high in Malaysia? Why do you think so?

-have you encounter any patient die from severe dengue due to delayed treatment?

-what do you think is the barrier for patients to seek treatment in time?
