# Supplementary material for: Self-care practices and health-seeking behaviours in patients with dengue fever: A qualitative study from patients’ and physicians’ perspectives
Source: PLoS Negl Trop Dis. 2023 Apr 27;17(4):e0011302. doi: 10.1371/journal.pntd.0011302 (PMC10168551; doi:10.1371/journal.pntd.0011302)
Supplement: S2 Interview — (DOCX) [file pntd.0011302.s002.docx]

**Topic guide for patients**

**Study title:** Exploring self-care practices and health-seeking behaviours of patients with dengue fever: A qualitative study from the perspectives of patients and doctors

Preamble:

• Ice-breaking session

• Explain purpose of session

• Interested in personal views and opinions

• No right or wrong answers

• Permission to refuse answer questions

• Views will be kept confidential

• Explain the necessity to audiotape (to help analyse your views in detail later on)

• Get demographic data and take electronic consent (for both interview and audiotaping)

• Any questions before we start?

1. Could you tell us more about your experience when having dengue fever?

-initial presentation: symptoms, duration

-how were you diagnosed?

-what happened during the consultation?

-any advice given by the doctor?

-continue follow up until recovery? (compliant/defaulted)

if not, why?

-Have you been admitted to the hospital?

1. What do you do at home after seeing doctor and diagnosed with dengue fever?

-Do you have any self-management theory?

-Have you heard of/tried any home remedies/traditional method? Explain briefly.

-Is that helpful/effective? In what ways?

-Do you monitor your symptoms at home?

If yes: how do you monitor?

If not: why not?

-Will you see doctor earlier than the daily follow up if you notice you are feeling sick? How do you decide to see doctor early?

-Is there anything that stop you from going to see the doctor? (barriers- eg. lack of knowledge, trouble from travelling and waiting, needed to work etc)

1. Can you describe the care that you received throughout the illness?

-how do you think about the care that you have received?

-can you please suggest ways to improve the healthcare experiences?

1. Have you ever heard of severe dengue/warning signs of dengue? (old terms including dengue haemorrhagic fever/dengue shock syndrome)

-if yes: how and what do you know about it?

-if not: do you think you need the information about it? Where do you think you can get the information from?

-is dengue consider a dangerous disease for you?

-how to you perceive the severity of dengue fever?

-do you know dengue can cause death?

-how do you think dengue causes death?

-do you think the chances of dying from dengue fever high or low?
