# Supplementary material for: Self-care practices and health-seeking behaviours in patients with dengue fever: A qualitative study from patients’ and physicians’ perspectives
Source: PLoS Negl Trop Dis. 2023 Apr 27;17(4):e0011302. doi: 10.1371/journal.pntd.0011302 (PMC10168551; doi:10.1371/journal.pntd.0011302)
Supplement: Attachment — Submitted filename: Replies to reviewers.docx [file pntd.0011302.s003.docx]

**Replies to reviewers**

**(the page and line number mentioned below are based on the tracked changes version of the manuscript)**

**Comment:**

Reviewer #1: The audience for this work would be extremely limited: as the health practices and societal beliefs presented in this paper would be mostly limited to Singapore medical practice, with limited generalizability beyond.

The methodology is also limited: limited number of participants and practitioners took part. There is no explanation of typical qualitative best practices such as evidence of interview saturation or conceptual overlap, rendering the findings thoroughly acquired and relevant.

**Reply:**

We have added explanation for our data saturation in ‘Participant recruitment’ subsection under Methods section:

We stopped recruitment when we reached data saturation during data collection and analysis for both patients’ and doctors’ interview. Data saturation was reached when there was no additional data found from both patients’ and doctors’ interview, and no new themes emerged. Additional one to two interviews were then conducted for both patient and doctor groups to ascertain data saturation. (line 124-128, page 6)

--------------------------------------------------------------------------------------------------------------------------------------

**Comment:**

Reviewer #2: Minor revisions suggested - Within the "data collection and instrument" section a literature review is mentioned very briefly, some additional information surrounding the literature review would be beneficial e.g., was this conducted by the authors? How many papers were reviewed? A bit more information about the interviews would also be good. It is stated the interviews were conducted on Zoom, were these with video on or off? Singular tense is used for the clinician interview, does this mean only one of the clinicians were interviewed and the remaining participated in the focus group? Would be good to clarify. Other than these points I think the study design (including the objective) was clear, the population well described and analyses deemed appropriate.

**Reply:**

We have provided additional information regarding the literature review:

The authors reviewed 13 articles focusing on the health belief, attitude, practice and experiences related to dengue fever to help develop the interview guides. (line 132-133, page 6)

We have also clarified the usage of video function during our interviews with Zoom:

Participants were given the option to turn their video off if they were not comfortable keeping their video on. (line 154-156, page 7)

The usage of focus group discussions (FGDs) and in-depth interview (IDI) for the clinicians have been clarified in the Results section:

A total of 13 patients were interviewed via IDIs and 11 doctors via FGDs and IDI (two FGDs consisting of five doctors per session; one doctor via IDI). (line 186-187, page 9)

--------------------------------------------------------------------------------------------------------------------------------------

**Comment:**

Reviewer #3: Methods and study population are appropriate. Data analysis process was clearly explained. No ethical concerns.

a. Participant recruitment: How was the number of patients interviewed determined? How many patients and doctors refused to participate and what were the reasons for non-participation?

**Reply:**

The recruitment of participants was stopped after we reached data saturation. Additional one to two interviews were conducted to ascertain data saturation. As mentioned above, we have added statements in “Participant recruitment” subsection under Methods to explain this:

We stopped recruitment when we reached data saturation during data collection and analysis for both patients’ and doctors’ interview. Data saturation was reached when there was no additional data found from both patients’ and doctors’ interview, and no new themes emerged. Additional one to two interviews were then conducted for both patient and doctor groups to ascertain data saturation. (line 124-128, page 6)

For the participant refusal, we have added the following information in the Results section:

Five patients did not pick up our phone calls during recruitment phase while another three patients refused to participate after we contacted them. We did not pursue the reasons for refusal. None of the doctors we approached refused participation. (line 187-190, page 9)

--------------------------------------------------------------------------------------------------------------------------------------

**Comment:**

b. Data collection and instrument, pg. 6: How long on average did the individual interviews and focus groups last? Did all 11 physicians participate in both IDIs and FGDs?

**Reply:**

We have added the information on duration of interviews in the “Data collection and instrument” subsection:

The mean duration of interviews were 36 minutes for IDIs (range: 21 to 55 minutes) and 53 minutes for FGDs (range: 51 to 55 minutes). (line 158-160, page 7)

Regarding the usage of FGDs and IDI for the clinicians, we have included the information in the Results section as mentioned above (line 186-187, page 9).

--------------------------------------------------------------------------------------------------------------------------------------

**Comment:**

Reviewer #1: Limited results presented; limited relevance to perhaps only dengue treatment and care within Singapore.

**Reply:**

Some of the themes are relatable universally such as use of complementary diet although the type of diet may differ according to regions. For example, papaya leaf extract is popular in India, Indonesia and Malaysia with studies from these countries on papaya leaf extract included in systematic review.

We have revised some sentences to improve on the generalizability of findings. For example:

Addressing complementary diets and practices in consultation, such as the papaya leaf extract, is important owing to their popularity in countries endemic for dengue such as India, Indonesia and Malaysia. (line 442-445, page 20)

Other issues highlighted such as gaps in communication between doctors and patients and childcare issue are also relatable to many regions.

**--------------------------------------------------------------------------------------------------------------------------------------**

**Comment:**

Reviewer #2: Minor revision suggested - I would like some more information about the sample. Specifically, it would be good to know how much time had it been since patients had experienced dengue. As noted in the methods section patients had to have received outpatient follow up for dengue within the past three months - but what was the actual amount of time since experiencing dengue of participants? Some extra descriptive statistics of demographic characteristics would also be useful e.g., Mean age. I thought the results themselves were presented really well and told a comprehensive narrative.

**Reply:**

We have included the additional information on mean age, and time lapse between onset of dengue and interview session in Table 1 (page 9-10).

--------------------------------------------------------------------------------------------------------------------------------------

**Comment:**

Reviewer #3: Findings are clearly presented.

a. Nice integration of the qualitative data in the Results section.

b. Pg. 8, Table 1

-	Were patients asked about their history of dengue fever infection? Health seeking behaviour and risk perception may be affected by a previous, mild case of dengue as patients may underestimate their risk for severe dengue and overestimate the effectiveness of home remedies in resolving dengue fever symptoms.

-	Suggest changing “Dengue Admission” to “Hospital Admission” since the parameter is whether or not the individual was admitted to the hospital.

**Reply:**

We have included information on history of dengue fever in Table 1. Two of our patients had prior history of dengue fever with no hospitalization. (page 9-10)

We have changed the term “Dengue Admission” to “Hospital Admission” in Table 1 as suggested.

--------------------------------------------------------------------------------------------------------------------------------------

**Comment:**

c. Pg. 9, Quotes

-	Could the sex of the patient be added as well, e.g., “Patient 3, 25-year-old female, not admitted”. It can be useful to see how men and women discuss the topics.

-	It would be nice to have the sex of the physician as well, but only if it does not increase the possibility that the physician participant could be identified as there is background information included in the manuscript (i.e., all are primary care physicians training in family medicine and years of practice are listed after the quote).

**Reply:**

We have included the sex of both patients and physicians in the quotes. We do not deem the sex as a potential identifier in our sample.

--------------------------------------------------------------------------------------------------------------------------------------

d. Theme 1: Use of complimentary diet for dengue

**Comment:**

-	Pg. 9, line 181: “leave” should be “leaf”

**Reply:**

We have corrected the spelling error. (line 208, page 11)

**--------------------------------------------------------------------------------------------------------------------------------------**

**Comment:**

-	Pg. 10: Were the patients asked if the doctor gave them a reason why they should not drink papaya leaf juice or crab soup? While some of the doctors stated that they might tell their patients there was no scientific evidence for a complementary diet, the quote from “Patient 13, 35 years old, admitted” (“And then I also kept quiet”) is a perfect example of how doctors shut down communication with patients.

**Reply:**

We have added a new quote under Results: Theme 1 (Use of complementary diet for dengue):

When asked if doctors gave them any reason not to take complementary diet such as papaya leaf juice and crabmeat soup, lack of scientific evidence was the reason quoted by doctors.

*“Two weeks later, when I follow up with doctor (a different doctor), I asked the doctor, “Why did the specialist stop me from drinking crabmeat soup?”*

*After that, he answered, “Maybe because there’s no research that showed crabmeat soup helps with dengue.”*

*Okay, I am okay with that, that’s what I understand.” (Patient 13, 35 years old female, admitted, had previous history of mild dengue)*

(line 237-245, page 12)

--------------------------------------------------------------------------------------------------------------------------------------

**Comment:**

-	Pg. 10, line 214: Suggest not mentioning “CAM” as complementary and alternative medicine is much broader than the health practices and health seeking behaviours that are the focus of this manuscript. If it is part of a direct quote, then put the quote in so you don’t have to describe CAM in the introduction.

**Reply:**

We agree with the suggestion and have replaced the term “CAM” with “complementary diet”. (line 250, page 12)

--------------------------------------------------------------------------------------------------------------------------------------

e. Theme 3: Symptom severity and social circumstances influence health-seeking

**Comment:**

-	Did patients mention cost as a barrier to follow-up visits? Costs could be financial (transportation, medical expenses not covered by the health system) and time away from other things such as work, school, family, etc.

**Reply:**

We went through our transcripts and coding again, and identified a quote on financial cost. We have included the quote in Results section and revised the introductory statement for the quotes. Additionally, we included another barrier for follow up visit on dislike of venepuncture:

Some patients who were not keen for daily follow up due to several reasons: financial cost (medical expenses), dislike of hospital visits in general, feeling physically fine, dislike for venepuncture, logistic hassle and long waiting hours in clinic. (line 352-355, page 16-17)

*“And also the cost, because I am paying also, I am paying a lot. So, like I feel I am ok, I feel my body getting better, why I have to go and pay every day that amount of money.” (Patient 1, 30 years old male, not admitted)* (line 363-365, page 17)

*“Of course, take blood test is not pleasant. You have to stick a needle through your skin, and then, it's not pleasant.” (Patient 5, 20 years old male, admitted after three days of outpatient follow up)* (line 369-371, page 17)

--------------------------------------------------------------------------------------------------------------------------------------

**Comment:**

-	Pg. 13: Not having someone to take care of children while the caregiver goes to the clinic has been identified as a major barrier to health seeking in general, especially for women, yet there is little in the dengue literature that addresses this issue. The authors could more fully discuss this issue in the Discussion. While not ideal, would video visits be an option for some of the patient follow-up care?

**Reply:**

We added a quote on this childcare issue in Results section – Theme 3 (Symptom severity and social circumstances influence health-seeking):

*“When I was diagnosed with dengue, my family from hometown…they came to my house, they took my two children, then they bring back to hometown. So, I was not looking after my children at that time, and I am with my wife only. My wife can take care of me and send me to hospital daily. But for others, it may be difficult for them to come to hospital if there is no one to send them, especially on weekends where there are no babysitters.” (Patient 11, 29-year-old male, not admitted)* (line 322-327, page 15)

We have expanded our discussion on this issue:

Of note, childcare responsibility is one of the barriers to healthcare seeking found in our study. Childcare responsibility is widely recognized as a barrier to healthcare access, particularly among women. Nair SK (2018) reported on health seeking behaviour for infectious diseases such as dengue whereby patients, especially women, did not access healthcare services despite recommendation from doctors, owing to familial responsibility. Interestingly, both patients who mentioned about childcare issue in our study are men, in contrast to what were commonly reported in the literature. Conventionally, men were seen as the breadwinners while the women were the homemakers. Men were perceived to have more time to care for themselves and have better access to healthcare. For our sample, this may be indicative of a changing gender role and sharing of childcare responsibility among parents especially in an urban setting like ours. Nevertheless, the neglect of proper care for acute illness as dengue, which can deteriorate rapidly, is worrying as observed in our study. Setting up a non-profit childcare centre at the healthcare centres to address the lack of childcare issue is an interesting endeavour, as demonstrated by Alvarez KS et al (2022). This would require facilities upgrade, integration of childcare service into the existing clinic workflow and engagement with community-based childcare organisations. (line 517-532, page 23-24)

Regarding the option of video visits, we commented on this in a later part of the discussion:

The concept of remote monitoring for dengue such as virtual consultation or mobile application for self-monitoring is interesting but the safety and efficacy of such intervention should be vigorously studied first before implementation. (line 552-555, page 25)

-------------------------------------------------------------------------------------------------------------------------------------

**Comment:**

-	Pg. 16, line 353: Define “FBC” in quote.

**Reply:**

We have defined “FBC” in the quote, which was actually referring to “Full blood count”. (line 413, page 19)

--------------------------------------------------------------------------------------------------------------------------------------

**Comment:**

Reviewer #1: The questionnaire/discussion guide was not shared--making conclusions that the authors came up with hard to interpret.

Several "conclusions" seemed to be perceptions of the authors, rather than necessary fact from the qualitative work.

**Reply:**

We will share the interview guides for patients’ and doctors’ groups as the supplementary materials.

--------------------------------------------------------------------------------------------------------------------------------------

**Comment:**

Reviewer #2: The conclusions are supported by the results, however when discussing the first theme the language surrounding taking the "middle-ground approach" did not make it clear that some doctors did take this approach (as mentioned in the results) so I would suggest revisiting this text to enhance clarity. Strengths and limitations are clearly described and the research's relevance is clear.

**Reply:**

We have revised the sentence to clarify that some of our doctors took this approach as well:

Perhaps, a more appropriate approach would be to take the ‘middle ground’ stand, as practised by some of our doctors in this study, where doctors can acknowledge the ‘no harm’ perception by patients but also inform them regarding the absence of benefit. (line 439, page 20)

--------------------------------------------------------------------------------------------------------------------------------------

Reviewer #3: Conclusions are supported by the data and relevance to good clinical outcomes is discussed.

a. Pg. 17

**Comment:**

-	line 360: “perspective” should be “perspectives”

**Reply:**

We have corrected the word “perspectives” in this manuscript and also checked the rest of the manuscript to ensure that the word is in plural form.

--------------------------------------------------------------------------------------------------------------------------------------

**Comment:**

-	lines 372 and 373: It wasn’t only doctors who noted that tension in the consultation could be created by physicians telling patients not to use the complementary diet; Patient 13 said they “kept quiet” after the doctor told them not to drink the crab soup or papaya juice. It would have been interesting to hear what the patient thought about that specific physician - did they trust them? did they feel they were being listened to?

**Reply:**

We have revised the sentence in the Discussion section to include the patient component in realizing the tension in consultation:

This may create tension in the consultation, as pointed out by some doctors and patients in our study. (line 437-438, page 20)

We have re-examined our recordings and transcripts, particularly of Patient 13, but we did not find any notable quotes to outline what patient thought of the doctor, or other doctors in general in regard to the communication on complementary diet.

-------------------------------------------------------------------------------------------------------------------------------------

**Comment:**

c. Pg. 19

-	line 411: The phrase “…made them understand better” is not clear nor an accurate summary of the two articles cited. Perhaps something like this clarifies the authors’ point: “The reason might be due to the stressful situation in the hospital that affected the patient’s reception and retention of information about the disease, whereas access to the information at home, away from the stressful hospital visit and feeling less fatigued allowed greater comprehension of the material.”

**Reply:**

We agreed the recommendation from the reviewer captures our point more clearly and have revised the sentence accordingly. (line 476-479, page 22).

**--------------------------------------------------------------------------------------------------------------------------------------**

**Comment:**

-	lines 420-421: Suggest deleting the sentence “While doctors’ main considerations…” as it repeats the information from the previous sentence.

**Reply:**

We have deleted the sentence.

------------------------------------------------------------------------------------------------------------------------------------

**Comment:**

d. Pgs. 19 and 21: Suggest not using “unique” as the context is more individual rather than being the only one of a kind. For pg. 19 (line 430), perhaps “individual circumstances” and for pg. 21 (line 458), use of the word “challenges” without a descriptor would be appropriate given the context of home monitoring and self-care practices is specified.

**Reply:**

We have replaced the word “unique” with “individual” as suggested:

Consideration of these other factors is important in doctor’s consultation with patient as the information conveyed by doctors would be more beneficial if they addressed the patient’s concern, belief and ~~unique~~ individual circumstances. (line 513, page 23)

We have deleted the word “unique” from the sentence:

To our knowledge, there is no qualitative study focusing specifically on the outpatient care setting for patients with dengue fever, which presents ~~unique~~ challenges for home monitoring and self-care practices. (line 561, page 25)

--------------------------------------------------------------------------------------------------------------------------------------

**Comments:**

Reviewer #2: Propose a grammar check throughout, for example, tenses need looking at as they sometimes switch between present and past tense, singular language is also often used when the plural is needed.

Reviewer #3: Minor grammatical issues:

1. The manuscript is well-written. However, a good read-through is needed to correct instances where a word should be plural but it is singular, and a very few that are plural but should be singular. Some examples are:

-	Abstract, lines 40 and 41: “doctor” should be “doctors” while “outpatients” should be “outpatient”

-	Results, line 211: “benefit” should be “benefits”

-	Results, line 258: “patient” should be “patients”

etc.

2. Abstract, line 32: Word “patients” missing after “… targeting dengue…”

3. Introduction

a. Pg. 3, line 59: delete the extra “0” in the 32 per 1000,000 population

b. pg. 4, line 75: What does “default follow up” mean?

**Response:**

We have screened the manuscript again and made the necessary corrections.

The sentence on “default follow up” has been rephrased for better clarity:

Sometimes patients delay seeking help or miss the follow up during the acute illness. (line 91, page 4)

--------------------------------------------------------------------------------------------------------------------------------------

**Comment:**

Reviewer #2: Overall, I thought this research was of extreme importance and believe it is novel in exploring dengue management in patients and doctors and identifying discrepancies between these populations thoughts surround health-seeking behaviour. I thought the narrative read really well - however, to make it even more well-rounded I would propose including in the introduction some information surrounding general dengue symptoms vs symptoms which are considered "more severe" to aid interpretation of the results from those who may be less familiar with dengue.

**Reply:**

We have added two more sentences in the second paragraph of the introduction to explain the symptoms of dengue better:

Dengue is usually suspected when a high fever is accompanied by symptoms such as headache, retroorbital pain, muscle and joint pain, nausea, vomiting or rash. (line 72-74, page 4)

Doctors usually watch out for warning signs for severe dengue during the follow up such as abdominal pain, persistent vomiting, bleeding gum or nose, blood in vomitus or stool, breathlessness, lethargy and confusion. (line 80-82, page 4)

--------------------------------------------------------------------------------------------------------------------------------------

**Comment:**

Reviewer #3: This is a nicely written manuscript on a topic that has not been deeply researched. The comparison of patient and physician perspectives on self-care practices and when to seek medical care for dengue fever is novel and helpful for planning health education interventions as strategies and materials need to be developed for both physicians and patients.

While the authors mention in the Discussion the need for communication training for physicians, this recommendation could be more strongly presented as the identified gaps between patients and physicians show an urgent need for better communication. A patient-centered care approach could be a useful framework for this recommendation.

Additionally, some mention of who is responsible for developing interventions to address the incomplete knowledge about dengue, dengue warning signs in particular, and follow-up care would support the findings. For example, questions such as what combination of verbal and visual information should be shared with patients? How should this verbal and visual information be provided – during the visit, through print materials such as pamphlets or online videos, or a combination of methods, among others, should be addressed in a health education intervention.

**Reply:**

We have revised the third paragraph of our discussion to include points on communication skills and patient education:

The gaps between information provision by doctors and knowledge retention by patients in our study highlight an urgent need for more effective communication strategies for outpatient dengue care. A patient-centred care approach, encompassing physical symptoms, personal preferences, values, cultural belief and socioeconomic conditions, is aptly for consultation of acute dengue illness. Supplementing the verbal consultation session with graphic chart may be useful to enhance patients’ comprehension of dengue fever, as practised by some doctors in our study. Visual tool has been shown to be a good supplement to verbal education. Written self-care advice are routinely provided to the patients in our setting but our study shows that it may not be adequate for information retention by patients. In the primary care setting, the follow up care should be utilized by doctors and patients to clarify any knowledge gap, with doctors taking a proactive role to allow time for questions. Implementing the teach-back method during consultation, in which the doctors ask the patients to repeat the information back to them to confirm understanding of information, can be a good strategy. Public health officials and clinical experts can improve on the existing dengue training modules by incorporating communication skill or a patient education module as the current modules generally focus more on disease management and rarely address patient communication aspects. (line 481-500, page 22-23).
