# Supplementary material for: Self-care practices and health-seeking behaviours in patients with dengue fever: A qualitative study from patients’ and physicians’ perspectives
Source: PLoS Negl Trop Dis. 2023 Apr 27;17(4):e0011302. doi: 10.1371/journal.pntd.0011302 (PMC10168551; doi:10.1371/journal.pntd.0011302)
Supplement: Attachment — Submitted filename: DeSMoS manuscript_PLOS NTD_editor tracked thorugh introduction.docx [file pntd.0011302.s004.docx]

**Self-care practices and health-seeking behaviours in patients with dengue fever: a qualitative study from patients’ and doctors’ perspectives**

**Short title: Self-care and health-seeking behaviours in dengue**

**Authors**

Wei Leik Ng^1*^, Jia Yong Toh^1¶^, Chirk Jenn Ng^1,3,4*^, Chin Hai Teo^1,2¶^, Yew Kong Lee^1,2&^, Kim Kee Loo^1&^, Haireen Abdul Hadi^1&^, Abdul Muhaimin Noor Azhar^5&^

**Author affiliations**

1 Department of Primary Care Medicine, Faculty of Medicine, Universiti Malaya, Kuala Lumpur, Malaysia.
2 UM eHealth Unit, Faculty of Medicine, Universiti Malaya, Kuala Lumpur, Malaysia.
3 SingHealth Polyclinics, Singapore.
4 Duke-NUS Medical School, Singapore.
5 Department of Emergency Medicine, Faculty of Medicine, Universiti Malaya, Kuala Lumpur, Malaysia.

***** Corresponding authors

Email: [wlng@ummc.edu.my](mailto:wlng@ummc.edu.my) (WLN), [ngcj@um.edu.my](mailto:ngcj@um.edu.my) (CJN)

^¶^These authors contributed equally to this work.

^&^These authors also contributed equally to this work.

# Abstract

**Introduction**: Outpatient management for dengue fever is the mainstay of treatment for most dengue cases. However, severe dengue can develop rapidly while patients are at home. Understanding the self-care practices and healthcare-seeking behaviours among dengue patients managed as outpatients will help improve the delivery of care to these patients.

**Objective**: This study aimed to explore the self-care practices, health-seeking behaviour and outpatient management of dengue fever from the perspectives of patients and primary care doctors.

**Methodology**: This was a qualitative study using in-depth interviews and focus group discussions, targeting dengue patients who received outpatient care and primary care doctors who cared for them. Patients and doctors shared their experiences and perception of self-care practices, decisions to seek urgent care and outpatient management of patients diagnosed with dengue fever. Data were coded and analysed using thematic analysis.

**Results**: 13 patients and 11 doctors participated. We discovered that the use of traditional remdies was common with patients perceiving no harm from it, while doctors did not see the benefit. Dengue patients’ knowledge of warning signs was inadequate despite the information being provided by doctors during follow up visits during their illness. We also find that symptom severity and social circumstances influence patients’ health-seeking behaviour, whereas most doctors only considered the presence of warning signs as factor influencing the behaviour. Regular outpatient follow up for dengue was perceived as inconvienent by patients. At the same time, there were practice variations in outpatient follow up for dengue.

**Conclusion**: There are gaps in patients’ knowledge on dengue fever and perceptions around self-care practices, health-seeking behaviour and outpatient management of dengue fever often differed between doctors and patients. Addressing these gaps in patient knowledge and distinct perceptions are needed to improve the safety and delivery of outpatient care for dengue patients.

**Keywords**: dengue, outpatient, self-care, health-seeking behaviour, primary care

# Author Summary

Dengue is a mosquito-borne viral disease that is endemic in many tropical and subtropical regions. Most dengue cases are self-limiting and can be managed as outpatient without hospitalization. Once diagnosed, patients are advised to return to the clinic daily for monitoring until they recover. However, some patients can deteriorate rapidly at home in between follow up and fail to recognize the deterioration. Poor self-management often leads to a delay in seeking help and, hence treatment; this has a significant impact on morbidity and mortality. This research provides insight into the self-care practices, health-seeking behaviour and outpatient management of dengue from the perspectives of patients with dengue and primary care doctors who manage them.

# Introduction

Globally, the incidence of dengue has increased in the recent few decades with cases reported in up to 130 countries (1). Americas, Southeast Asia and Western Pacific regions are mostly affected with Southeast Asia and Western Pacific region bearing 70% of the global dengue burden (2, 3). It was estimated that worldwide, more than 9,000 dengue deaths occurred annually between 1990 and 2013 (1). Southeast Asia, including Malaysia, reported some of the highest dengue mortality rates in the world [1]. Dengue fever has been endemic in Malaysia since the 1990s and incidence has increased dramatically from 32 cases per 100,000 in 2000 to 361 cases per 100,000 population in 2014 (4).

Outpatient management of dengue fever by primary care doctors is the mainstay of treatment as most dengue cases are mild and self-limiting (5). Dengue is usually suspected when a high fever is accompanied by symptoms such as headache, retroorbital pain, muscle and joint pain, nausea, vomiting or rash (4). There are three phases of illness in dengue fever: febrile phase, critical phase and recovery phase. The critical phase typically comes after 3 days of fever and is marked by a rapid drop in body temperature; this may be misconstrued as a recovery although patients may actually develop bleeding, rapid loss of fluid or organ damage during this phase (4, 5). Once diagnosed with dengue fever, patients are usually expected to come back to the clinic regularly to monitor clinical symptoms and blood profile to catch any deteriorating signs for early intervention (4). Doctors usually watch out for warning signs for severe dengue during the follow up such as abdominal pain, persistent vomiting, bleeding gum or nose, blood in vomitus or stool, breathlessness, lethargy and confusion (4). Optimal outpatient clinical management is crucial to reduce morbidity and mortality in dengue infection.

Effective outpatient management depends on appropriate self-care practices and timely health-seeking, especially when the patients’ condition deteriorates. Dengue patients managed as outpatients are usually advised to seek medical help immediately when dengue warning signs, emerge. This usually occurs during the critical phase of dengue and patients with comorbidities such as diabetes mellitus and hypertension are at higher risk of developing severe dengue (6, 7). Sometimes patients delay seeking help or miss their regular follow up visits during the acute illness. Reasons for this include poor comprehension of health advice provided by doctors, poor self-care and personal health beliefs (8-11).

There is a dearth of research on self-care practices and health-seeking behaviour of patients with dengue, especially in Southeast Asia; both depend on local socio-cultural contexts and health systems. In this study, we aimed to explore the self-care practices, health-seeking behaviour and outpatient management of dengue fever from the perspectives of patients and primary care doctors. The findings from this study would give us an insight on how to intervene and provide better care for patients with dengue in the outpatient setting.

# Methods

## Study design

This study used a qualitative methodology to explore patients’ and doctors’ experiences in dengue management. A descriptive interpretive approach was used to describe and interpret the collective experience of the participants in the health setting (12).

## Participant recruitment

The participants of this study consisted of patients and primary care doctors at University of Malaya Medical Centre (UMMC). UMMC is located in a central urban area in Malaysia with a high annual incidence of dengue.

The patients were identified from the Department of Primary Care Medicine, University of Malaya Medical Centre (UMMC) electronic medical records. Once identified, patients were screened for eligibility using the following criteria: i) aged 18 years old and above, ii) diagnosed with dengue fever with confirmatory serology test, and iii) received outpatient follow up for dengue within the past three months. The patients were selected purposively based on their age, ethnicity and severity of dengue (not admitted; admitted) to achieve maximal variation in terms of their self-care needs and challenges faced.

The doctors were identified from the primary care clinic in UMMC, with eligibility being having experience in managing outpatient dengue cases. They were the family medicine trainees based in the clinic.

The eligible participants were contacted via phone and recruited by researchers and research assistant. Appointments were set for the participants to be interviewed online via Zoom. The participants were reimbursed RM50 (approximately USD 12) for their effort and time spent on the interview.

We stopped recruitment when we reached data saturation during data collection and analysis for both patients’ and doctors’ interview. Data saturation was reached when there was no additional data found from both patients’ and doctors’ interview, and no new themes emerged. Additional one to two interviews were then conducted for both patients and doctors group to ascertain data saturation.

## Data collection and instrument

Separate interview guides for patients and doctors were developed based on health behaviour theories, namely the health belief model (13) and theory of planned behaviour (14), and literature review. The authors reviewed 13 articles focusing on the health belief, attitudes, practices and experiences related to dengue fever to help develop the interview guides. They were used to facilitate the semi-structured interviews. During the interviews, doctors were asked to describe their experiences when treating dengue patients, challenges that they encountered during the consultation and subsequent follow up with dengue patients, their practice in dengue case management and experiences in handling difficult dengue cases; while the patients were asked to recall their experiences throughout the illness, from initial presentation, diagnosis, follow up till recovery. In particular, their experiences during consultations with doctors, self-management practices at home, and health service utilization were explored. In addition, patients were asked about their care and suggestions to improve their healthcare experiences. In-depth interviews (IDIs) were conducted for patients while both focus group discussions (FGDs) and in-depth interview were used for doctors.

Interviews were conducted by WLN, JYT and CJN. WLN and CJN were clinicians and medical lecturers in Department of Primary Care Medicine, UMMC. JYT was the research assistant for the study. WLN, CJN and JYT held medical degrees. Additionally, CJN had a PhD and master’s degree in family medicine while WLN had a master’s degree in family medicine. Interviews were conducted in either Malay or English depending on the patient’s preferred language as all researchers were fluent in both languages.

Due to the COVID-19 pandemic, the study was conducted using an online platform, specifically Zoom (Zoom Video Communications, California). To ensure confidentiality, all teleconference sessions were only accessible using a password. The participant information sheet and electronic consent form (Google Form) for participation were sent to the participants through email/WhatsApp and the consents were obtained before the interview. Participants were given the option to turn their video off if they were not comfortable keeping their video on. All interviews were recorded using Zoom’s function after consents were obtained. No one else besides the participant and researchers (WLN, JYT and CJN) was present during the interview. Field notes were taken by researchers during the interviews. The mean duration of interviews was 36 minutes for IDIs (range: 21 to 55 minutes) and 53 minutes for FGDs (range: 51 to 55 minutes).

## Data analysis

The audio recordings were transcribed verbatim and anonymized. The transcripts were analyzed independently by two researchers (WLN and JYT) using the thematic approach, which was first by reading and re-reading the transcripts to familiarize themselves with the content, followed by open coding and subsequently axial coding. Triangulation was done by comparing the findings between the doctors and patients to identify similarities or differences in their perceptions. The researchers met to compare and discuss the coding, and reconcile any discrepancies via a team discussion. The researchers critically examined and reflected on their roles throughout the study to reduce potential biases during the interviews and data analysis. ATLAS.ti version 8 software was used to manage the data. Transcripts were analyzed in either Malay or English as all researchers were familiar with these languages; participant quotes in Malay were translated into English for reporting.

## Ethical consideration

This study was approved by the University of Malaya Medical Centre (UMMC) Ethics Committee (MREC-20201021-9157). All participants provided written informed consent prior to enrolment in the study.

# Results

A total of 13 patients were interviewed via IDIs and 11 doctors via FGDs and IDI (two FGDs consisting of five doctors per session; one doctor via IDI). Five patients did not pick up our phone calls during the recruitment phase while another three patients refused to participate after we contacted them. We did not pursue the reasons for refusal. None of the doctors we approached refused participation. These interviews were conducted between 8th December 2020 to 5th June 2021.

For the patients, most of them were working adults with no comorbidities. All of them had received outpatient follow up for dengue fever and a few of them had history of hospitalization due to the worsening of dengue fever. All doctors in the interview were primary care doctors with experience in managing dengue fever.

The demographic details of the study sample are shown in Table 1.

**Table 1: Demographic summary of the participants**

| **Patients (N=13)** | |
| --- | --- |
| **Parameters** | **Description** |
| Age | 19 – 58 years old (mean age: 35, standard deviation: 13) |
| Gender | 9 males, 4 females |
| Ethnicity | 5 Bumiputera, 5 Chinese, 2 Indian, 1 Syrian |
| Comorbidities | 10 without comorbidities, 3 with comorbidities such as hypertension, diabetes mellitus, gout and iron deficiency anaemia) |
| Hospital admission | 4 were admitted for dengue while 9 were not. |
| Mean time lapse between onset of recent dengue and interview session | 46 days (range: 8 to 77 days) |
| History of dengue (prior to current episode) | Only two participants had previous episodes of dengue at three years and five years ago, respectively. They only received outpatient care with no hospitalization during the previous episodes. |
| **Doctors (N=11)** | |
| **Parameters** | **Description** |
| Age | 32 – 35 years old (mean 34, standard deviation 1) |
| Gender | 6 males, 5 females |
| Ethnicity | 1 Bumiputera, 8 Chinese, 2 Indian |
| Years of practice | 5 – 10 years |
| History of dengue fever | None had ever been personally infected with dengue fever |

Four themes emerged from our interviews with both patients and doctors.

## Theme 1: Use of complementary diet for dengue

Dietary modification was the main change during self-care at home. Increasing fluid intake was the main modification made. The modification in fluid intake was primarily influenced by doctors’ advice. The choice of fluid varied from warm water, lemon water, isotonic drink, coconut water, soup to barley water.

*"The doctor informed me, ok for dengue all I need is to drink more water, there is no cure for dengue, ok, since there is no cure and then good enough…" (Patient 6, 58 years old male, not admitted)*

Complementary diet such as papaya leaf extract, guava juice and crabmeat soup was commonly used in our patients, out of which papaya leaf juice was the most frequently mentioned. The use was influenced by family, friends, community (neighbour) and social media. Patients perceived no harm from taking them, regarding them as part of their usual diet. Patient 3 also mentioned that the desperation to get well again influenced her to try a complementary diet, knowing that there was no definitive treatment for dengue.

*"They also made like, you know, moms being moms, they make weird type of soup for you to drink, like crab soup I don't know what the hell is that? So basically that and I also, I guess they also heard from other people that, you know, if you are having dengue, you have very weak immune system, you should be taking things like papaya extract pill." (Patient 3, 25 years old female, not admitted)*

*“I only believe in like scientific evidence, obviously, I don't believe in all of these. But I guess like, you know, when you are desperate, you just want to try everything to be well again, I mean, there is no cure for dengue.” (Patient 3, 25 years old female, not admitted)*

Some patients received advice from doctors not to take such complementary diet. Patient 1 commented that his doctor did not address any dietary measures apart from increasing fluid intake.

*"The doctor asked me to drink, to not drink papaya leaf juice, the extract, yes. She told me not to do that, because it was very common among the patients. But my parents ended up giving it to me, but I did, I said I didn't want to, but I still drink it anyway." (Patient 5, 20 years old male, admitted)*

*"* *They (doctors) didn’t mention eating, they didn’t tell me eat this, don’t eat that. Nothing, just drink water, that’s it." (Patient 1, 30 years old male, not admitted)*

*“Recently I got admitted, the specialist, he mentioned to me don't drink crab soup, papaya juice. And then I also kept quiet, don’t know the reason, because they are normal food only, to me they are not any medicine” (Patient 13, 35 years old female, admitted,* *had previous history of mild dengue)*

When asked if doctors gave them any reason not to take complementary diet such as papaya leaf juice and crabmeat soup, lack of scientific evidence was the reason quoted by doctors.

*“Two weeks later, when I follow up with doctor (a different doctor), I asked the doctor, “Why did the specialist stop me from drinking crabmeat soup?”*

*After that, he answered, “Maybe because there’s no research that showed crabmeat soup helps with dengue.”*

*Okay, I am okay with that, that’s what I understand.”* *(Patient 13, 35 years old female, admitted, had previous history of mild dengue)*

The doctors in our interview generally took a more middle-ground approach when it came to complementary diet. They perceived there were no benefits from the additional dietary measures but they did not explicitly forbid patients from taking them. Doctor-patient relationship was also a consideration. Doctor 1 mentioned that it is important to respect the patient's belief on complementary diet provided it caused no harm to maintain a good patient-doctor relationship. He further explained that if doctors did not respect them, it would cause a barrier between them, making it difficult to develop trust with patients for future management.

*“I’ll ask what they take. If as long, I think it is safe, and it has some fluid inside it, like soup, papaya juice, because all these I will tell them that definitely from research is not proven. But as long is not harmful for them, and they are drinking something, so I would say, I would be clear to them, I would say it doesn’t have any beneficial thing, but at the same time if let say if it’s like harmful or anything then I will stop them from taking.” (Doctor 10, male, 6 years of experience)*

*“I mean the behaviour is not harmful for them, we should respect, because, if we want to change one person behaviour, then it might affect the relationship between us with the patient, and is hard for us to continue follow up with them.... But then once we change their perception on this traditional medication we said it might cause harm to you, then their relationship with us will start to have some barrier then.” (Doctor 6, male, 5 years of experience)*

## Theme 2: Gaps in information provision and patients’ knowledge

There were gaps in the patients’ knowledge of the dengue warning signs and different phases of illness. When specifically asked about the warning signs of dengue that they watched out for during their dengue fever episode, most patients mentioned vomiting, bleeding and diarrhoea. Some participants did not remember any warning signs being advised when discharged for home monitoring as outpatient management. Out of all participants, only one participant mentioned dengue fever causing death when asked about her understanding of the severity of dengue fever.

*"Ya, they told me the dangerous signs, ya, like diarrhoea, bleeding, if throwing up, ya, I have to go to hospital. But other than that, no, just drink water." (Patient 1, 30 years old male, not admitted)*

*“When entering critical phase I was shocked, because I did not have fever, and then doctor told me actually I was in critical phase. We thought if we stay at home, we feel no more fever, we are getting better.” (Patient 13, 35 years old female, admitted, had previous history of mild dengue)*

Patient 7 did not seek immediate treatment despite persistent vomiting because she felt she had nothing serious based on what the doctor had said during her initial first visit to the clinic.

*“I keep on vomit every five minutes…. I just rest, I just keep drink a lot of water. But after I drink water, I still vomit, the water out, yeah. But I just have to wait for the next day…….. because I went to the clinic (on earlier visit) and then the doctor said I have nothing serious. So, I didn't go to the hospital.” (Patient 7, 19 years old female, admitted)*

Doctors in our interview tailored information on danger signs and dengue phases based on patients’ knowledge and ability to understand. Doctor 4 would explain the different dengue phases to her patients, while Doctor 3 thought explaining dengue phases to patients was "too much" for them to retain the information. Regarding patients’ knowledge of dengue, Doctor 11 felt that most patients were quite knowledgeable about dengue already before seeing doctors.

*"Usually once confirmed with dengue, I will show them the dengue phase chart, then it will show like the febrile phase, the critical phase and also the recovery phase. I will let them know actually where exactly they are in, and then what is the timing have to watch out, watch out for the, for the critical phase all that, and then will inform them about the importance of monitoring with the FBC (full blood count).” (Doctor 4, female, 10 years of experience)*

*"I didn't go to the whole three phase thing; I think it's a bit too much for the patient. So, I’ll just state down what you need to look out for, all the warning, there's only a few warning signs so it’s easy to remember." (Doctor 3, female, 10 years of experience)*

*"I think most of our patients have at least a bit information about dengue even before they came to see the doctor, they already know that there are some sorts of dengue haemorrhagic fever, something like that, they know few things already. We just emphasize the danger of it" (Doctor 11, female, 9 years of experience)*

## Theme 3: Symptom severity and social circumstances influence health-seeking

For patients, symptom severity and social circumstances influenced their decision to seek help. Patients’ decision to seek immediate help was influenced more by the severity of symptoms they experienced and their social circumstances rather than their knowledge of the warning signs. Patient 2 had postponed his plan to seek assessment in the hospital although he was feeling unwell with dizziness because there was no one to take care of his children at home. Children’s caregiving issue regarding health-seeking was also raised by Patient 11.

*“I really wanted to go, hospital. But nobody was at home to take care of my children that time. Wife was working.” (Patient 2, 36 years old male, not admitted)*

*“When I was diagnosed with dengue, my family from hometown…they came to my house, they took my two children, then they bring back to hometown. So, I was not looking after my children at that time, and I am with my wife only. My wife can take care of me and send me to hospital daily. But for others, it may be difficult for them to come to hospital if there is no one to send them, especially on weekends where there are no babysitters.” (Patient 11, 29-year-old male, not admitted)*

Perceived severity was more based on clinical symptoms experienced by the patients (e.g. pain level, general health status) rather than a systematic checklist of all warning signs (like what doctors went through when they saw a dengue patient). For example, a patient would go to the emergency department immediately if his headache and eyeballs got really painful. Another patient went to the emergency department due to unbearable gastric pain but he did not recognise this as a dengue warning sign.

*"Maybe if my headaches or my eyeballs really painful, something is not right. But if the pain is only very mild, then not a necessity for me to go to hospital. I mean it’s our own body, we know how serious it is” (Patient 6, 58 years old male, not admitted)*

How the patients decided on seeking immediate care was different from our doctors’ assumption that patients already had the necessary knowledge to make decisions about when to seek immediate help. Doctors generally advised patients to come immediately to seek treatment when they developed warning signs. Doctor 2 said he did not face any resistance from patients when he advised patients to come immediately to hospital because the patients would already know the warning signs.

*"Usually no resistance from most of the patients, because seems like everyone has like the same baseline knowledge about dengue, and it’s quite acceptable that level of knowledge." (Doctor 2, male, 7 years of experience)*

## Theme 4: ‘Troublesome’ dengue follow ups

In regard to perception of outpatient management of dengue fever, which usually entailed regular, daily follow up in primary care clinics, patients appeared unclear about the reason for regular daily follow up. Some patients questioned the rationale of regular follow up when they felt they were able to assess their condition by themselves. Some patients were not keen on daily follow up due to several reasons: financial cost (medical expenses), dislike of hospital visits in general, feeling physically fine, dislike of venepuncture, logistic hassle and long waiting hours in clinic.

*“I’m not too sure what is the frequency of the visit. So, I just follow advice by the doctor” (Patient 10, 47 years old male, not admitted)*

*“Yes, I actually was thinking why can’t I come back like maybe every two days or more that. I only come back daily if like you know, they tell me the red flags that I should be aware of like ok maybe you get this this this, then you come back, you know, but then otherwise maybe after two days you repeat blood again. You know, I thought it could have been done like that." (Patient 3, 25 years old female, not admitted)*

*“And also the cost, because I am paying also, I am paying a lot. So, like I feel I am ok, I feel my body getting better, why I have to go and pay every day that amount of money.”* *(Patient 1, 30 years old male, not admitted)*

*“To me the very troublesome and cumbersome thing was actually having to go back every day for blood test. I actually was thinking why can’t I come back like maybe every two days or more” (Patient 3, 25 years old female, not admitted)*

*“Of course, take blood test is not pleasant. You have to stick a needle through your skin, and then, it's not pleasant.” (Patient 5, 20 years old male, admitted after three days of outpatient follow up)*

Lack of continuity of care in a public government clinic was also one of the factors mentioned by patients as the reasons they found daily follow up for dengue unpleasant.

*“That’s why I decided to go to clinic* (private general practitioner clinic)*. Because at least is one doctor. I don’t wanna see every day a different doctor, every day I need to tell them what happened, they asked the same question. You should read the report, know what happened to me. I am not gonna tell you every time.”* *(Patient 1, 30 years old male, not admitted)*

Among the doctors, we also noticed practice variation in the follow up regime. Consideration factors here included comorbidities, current dengue phases (febrile, critical or recovery), confirmed dengue serology, patients' clinical and vital signs, history of past dengue infection and distance from the hospital. Frequency of outpatient follow up was one area where they felt the national clinical practice guideline did not address well. There was no clear-cut guide on monitoring dengue patients as outpatient apart from watching out for warning signs. Outpatient management of dengue patients by doctors were guided by personal clinical experience, phase of dengue illness and clinical parameters. Generally, the doctors followed up their dengue patients daily but would space out the follow up at variable intervals when patients were in recovery phase.

*" I don't think is stated in the dengue CPG (clinical practice guideline) like how frequent we need to monitor. what I could recall is from, I think our houseman time where, depends on the hospital that we work, or the clinic that we work, we have a guideline, then we follow that and still carry on until today." (Doctor 1, female, 10 years of experience)*

*"It depends on which stage the patient is in. So, let say if the patient is in febrile phase, then we might be seeing them more often also. but if the patient is already in recovery phase, and then the platelet is ok, more than 100 then maybe will see them longer, maybe 5 days after, 1 week." (Doctor 7, male, 7 years of experience)*

Generally, the doctors in our study thought most patients were compliant with regular follow up because they understood the severity of dengue fever and were afraid of the complication.

*"I think when comes to dengue I think they are a bit worried, so I think they are compliant when comes to dengue, when I’ll tell them about the complications of dengue, what can happen. So, when you tell the complication and you include death, they will be a bit careful on that" (Doctor 10, male, 6 years of experience)*

Weekend was also an important factor in decision for outpatient monitoring. Doctors would be more prone to skip a day or two of monitoring over the weekend if patients were stable, especially in recovery phase. Discontinuity of care and rejection by emergency department (ED) were cited as problems associated with weekend monitoring of outpatient dengue cases.

*"Yeah, especially towards the weekends. Like when it, when they come over on Friday and they need to continue daily monitoring on Saturdays and Sundays. And there are changes to where they can go to. That becomes a bit difficult for us cause sometimes you say come to ED, but then ED does not accept them, so, that, that becomes a hassle for us to find out and also for the patients because they don't know where to go." (Doctor 2, male, 7 years of experience)*

*"Sometimes when I think the FBC (full blood count) was quite stable, and the patient was quite stable, sometimes because of the weekends delay, I just proceed to Monday” (Doctor 1, female, 10 years of experience)*

Weekend factor was also raised by patients concerning children’s caregiving. As mentioned in the quote by Patient 11 in Theme 3: Symptom severity and social circumstances influence health-seeking, parents might find it harder to look for babysitters during the weekend, and consequentially, making it difficult for them to come for follow up during the weekend.

# Discussion

In our study, we discovered four themes regarding the self-care practices and outpatient dengue management from patients’ and doctors’ perspectives, which are 1) use of complementary diet for dengue, 2) gaps in information provision and patients’ knowledge, 3) symptom severity and social circumstances influence health-seeking, and 4) ‘troublesome’ dengue follow ups. While there were similarities in their perspectives, we also noticed some mismatch between these two groups in certain aspects.

We noticed an interesting difference in dietary practices perceived and adopted by patients and those advised by doctors in our study. While patients generally saw complementary diet such as papaya leaves as normal diet with ‘no harm’, doctors thought of it more as diet with ‘no benefit’. Thus, while patients wanted to have more information about diet that would help improve dengue symptoms even if they were not proven, they experienced discouragement from doctors not to take such a complementary diet. In such scenario, doctors’ attitude was that if the complementary diet was not proven, i.e ‘no benefit’, they discouraged patients from taking it. This may create tension in the consultation, as pointed out by some doctors and patients in our study. Perhaps, a more appropriate approach would be to take the ‘middle ground’ stand, as practised by some of our doctors in this study, where doctors can acknowledge the ‘no harm’ perception by patients but also inform them regarding the absence of benefit. The patients can then decide for themselves on complementary diet. This of course applies only to dietary practices which do not show harm. Addressing complementary diets and practices in consultation, such as the papaya leaf extract, is important owing to their popularity in countries endemic for dengue such as India, Indonesia and Malaysia (15). It was shown that more than 80% of patients with dengue fever in Malaysia practised some form of traditional or complementary medicine (16, 17). Liu H et al showed that up to 40% of the patients with suspected dengue fever practised self-medication and traditional medication without seeking treatment in hospital when having fever (18). Another point to take home is doctors need to understand the desperation that influences patients to resort to alternative treatment. The mainstay of treatment for dengue is supportive with fluid therapy, not curative. The absence of definitive treatment and perceived ineffectiveness of conventional treatment are important factors that drive patients to look for alternative treatments (11, 19). Tackling this common issue in consultation may potentially avoid delayed healthcare seeking due to self-medication with complementary diet.

Of the gaps in information provision and patients’ knowledge of dengue fever, we found that the knowledge of dengue fever among patients was still lacking despite coming regularly for their follow up, particularly in the scope of warning signs and stages of dengue fever. Similar to our study, a previous study in Malaysia demonstrated that the general public could identify well the common symptoms of dengue fever such as high-grade fever, headache, arthralgia and myalgia but relate less to symptoms indicative of warning signs such as rapid breathing, restlessness and severe abdominal pain (20). This was one area where we found some mismatches between doctors and patients. Our doctors in this study felt that patients’ knowledge was generally adequate although this may not be true from our findings. We also noticed that while doctors generally covered the important warning signs in their advice to patients, that information was not retained well in our patients. Some patients totally could not recall any warning signs that have been told by the doctors. Several patients factors could lead to failure of transfer of knowledge from doctors to patients, such as patient distress, poor health literacy and misinformation (21). Patient distress is an important factor in dengue fever as patients may be generally unwell during the clinic visit, compounded by the waiting time and the distress associated with navigating the healthcare system. Patients tended to remember final instructions which addressed follow up instead of a long list of information that had been conveyed earlier (22). On the other hand, lack of time and poor communication skills by the doctors could also contribute to the poor transfer of knowledge to patients during consultation (21). Previous studies showed that verbal teaching and discussion during consultation sessions were less effective than computer technology or online information (21, 23). The reason might be due to the stressful situation in the hospital that affected the patients’ reception and retention of information about the disease, whereas access to the information at home, away from the stressful hospital visit and feeling less fatigued, allowed greater comprehension of the material(21, 24). The gaps between information provision by doctors and knowledge retention by patients in our study highlight an urgent need for more effective communication strategies for outpatient dengue care. A patient-centred care approach, encompassing physical symptoms, personal preferences, values, cultural beliefs and socioeconomic conditions, is aptly for consultation of acute dengue illness (25). Supplementing the verbal consultation session with graphic chart may be useful to enhance patients’ comprehension of dengue fever, as practised by some doctors in our study. Visual tool is a good supplement to verbal education (26). Written self-care advice is routinely provided to the patients in our setting but our study shows that it may not be adequate for information retention by patients. .In the primary care setting, the follow up visits should be utilized by doctors and patients to clarify any knowledge gap, with doctors taking a proactive role to allow time for questions. Implementing the teach-back method during consultation, in which the doctors ask the patients to repeat the information back to them to confirm understanding of information, can be a good strategy (27). Public health officials and clinical experts can improve on the existing dengue training modules by incorporating communication skill or a patient education module as the current modules generally focus more on disease management and rarely address patient communication aspects.

When it came to decision to seek immediate or urgent care, we noticed that what influenced patients to seek help (symptom severity and social circumstance) might not be what the doctors were expecting (danger signs). There is a lack of studies to investigate the factors influencing decision to seek immediate care among patients with confirmed dengue fever under outpatient care. However, a recent systematic review of factors influencing healthcare seeking in patients with dengue revealed findings similar to what we uncovered in our interviews. Among the similar factors we uncovered included individual (such as personal health belief, risk perception), interpersonal (social support) and organizational factors (access to healthcare: inconvenient operating hours, long waiting time, oversaturated emergency services) (28). Consideration of these other factors is important in doctors’ consultation with patients as the information conveyed by doctors would be more beneficial if they addressed the patients’ concern, belief and individual circumstances (29). Doctors do not identify what patients consider as important sometimes, potentially leading to varied healthcare-seeking behaviours among patients managed as outpatient for dengue fever.

Of note, childcare responsibility is one of the barriers to healthcare seeking found in our study. Childcare responsibility is widely recognized as a barrier to healthcare access, particularly among women (30-32). Nair SK (2018) reported on health-seeking behaviour for infectious diseases such as dengue whereby patients, especially women, did not access healthcare services despite recommendation from doctors, owing to familial responsibility (32). Interestingly, both patients who mentioned childcare issue in our study are men, in contrast to what was commonly reported in the literature. Conventionally, men were seen as the breadwinners while women were the homemakers. Men were perceived to have more time to care for themselves and have better access to healthcare. For our sample, this may be indicative of a changing gender role and sharing of childcare responsibility among parents, especially in an urban setting like ours. Nevertheless, the neglect of proper care for acute illnesses such as dengue, which can deteriorate rapidly, is worrying as observed in our study. Setting up a non-profit childcare centre at the healthcare centres to address the lack of childcare issue is an interesting endeavour, as demonstrated by Alvarez KS et al (2022) (31, 33). This would require facilities upgrade, integration of childcare service into the existing clinic workflow and engagement with community-based childcare organisations (33).In this study, we also uncovered concerns in both patients’ and doctors’ groups regarding the regular follow up needed for dengue fever. Daily or more frequent follow up is recommended for patients receiving outpatient care for dengue fever (4, 5). Our patients did not really appreciate the significance of daily follow up to monitor for warning signs, particularly during the critical phase. Compliance with the regular follow up was still there mainly due to adherence to doctor’s instruction. We also discovered organizational issues related to daily follow up that can be improved in the primary care setting: long waiting time, discontinuity of care and inadequate operating hours, particularly over the weekend. The weekend factor is something that needs to be tackled as it also influences how our doctors dictate the frequency of outpatient follow up for dengue patients. Similar to what was previously reported, our doctors practised an instinctive clinical approach to manage outpatient based on individual judgement and experiences, citing the inadequacy of current guidelines to tackle the ‘real-world’ situation (34). WHO guideline recommended “daily follow up for all patients except those who have resumed normal activities or are normal when the temperature subsides” (35). On the other hand, our local Malaysia dengue guideline recommended daily monitoring during febrile phase (more frequently towards late febrile phase), at least twice daily during critical phase and daily (or more frequently as indicated) during the recovery phase (4). The practices we observed in our interviews with doctors did not conform to these guidelines. The practicality of asking patients to come twice to clinic for follow up in the same day is also a consideration. The concept of remote monitoring for dengue such as virtual consultation or mobile application for self-monitoring is interesting but the safety and efficacy of such intervention should be vigorously studied first before implementation. We recommend a more pragmatic approach to decide on the frequency of follow up to be included in the guideline, tailored to the clinical phases of dengue, individual circumstances and healthcare setting.

## Strength and limitations

To our knowledge, there is no qualitative study focusing specifically on the outpatient care setting for patients with dengue fever, which presents challenges for home monitoring and self-care practices. We also explored this topic from both the perspectives of doctors and patients, allowing us the opportunity to compare the similarities and mismatched practices between these two groups.

However, we recruited our participants only from a single health centre in an urban area in Malaysia and these findings are only representative of an urban, more affluent setting. There may also be recall bias among our participants as some of the interviews were not conducted immediately after their illness.

# Conclusion

There are gaps in patients’ knowledge of dengue and mismatches between doctors and patients regarding self-care practices, health-seeking behaviour and outpatient management of dengue. There is a need to improve and personalize outpatient care delivery for dengue patients safely, owing to the variability in individual perception of the diseases, practice variations among doctors and organizational factors.

**Declarations of conflicting interests:** The authors have declared that no competing interests exist.

**Funding:** The authors (WLN, JYT, CJN, CHT, YKL, KKL, HAH, AMNA) disclosed receipt of the following financial support for the research: This work was supported by the University of Malaya Impact-Oriented Interdisciplinary Research Grant Programme (grant number: IIRG002B-2020HWB, URL: <https://umcms.um.edu.my/sites/research-cluster-office-v2/impact-oriented-interdisciplinary-research-grant-iirg-programme>). The funders had no role in study design, data collection and analysis, decision to publish, or preparation of the manuscript.

# References

1.	Stanaway JD, Shepard DS, Undurraga EA, Halasa YA, Coffeng LE, Brady OJ, et al. The global burden of dengue: an analysis from the Global Burden of Disease Study 2013. Lancet Infect Dis. 2016;16(6):712-23.

2.	Bhatt S, Gething PW, Brady OJ, Messina JP, Farlow AW, Moyes CL, et al. The global distribution and burden of dengue. Nature. 2013;496(7446):504-7.

3.	Guo C, Zhou Z, Wen Z, Liu Y, Zeng C, Xiao D, et al. Global Epidemiology of Dengue Outbreaks in 1990-2015: A Systematic Review and Meta-Analysis. Front Cell Infect Microbiol. 2017;7:317.

4.	Malaysia MoH. Clinical Practice Guideline of management of dengue infection in adults. 3rd edition ed. Putrajaya, Malaysia: Malaysia Health Technology Assessment Section; 2015.

5.	Lum L, Ng CJ, Khoo EM. Managing dengue fever in primary care: A practical approach. Malays Fam Physician. 2014;9(2):2-10.

6.	Rathore AP, Farouk FS, St John AL. Risk factors and biomarkers of severe dengue. Curr Opin Virol. 2020;43:1-8.

7.	Sangkaew S, Ming D, Boonyasiri A, Honeyford K, Kalayanarooj S, Yacoub S, et al. Risk predictors of progression to severe disease during the febrile phase of dengue: a systematic review and meta-analysis. Lancet Infect Dis. 2021;21(7):1014-26.

8.	Ang KT, Rohani I, Look CH. Role of primary care providers in dengue prevention and control in the community. Med J Malaysia. 2010;65(1):58-62.

9.	Elsinga J, Lizarazo EF, Vincenti MF, Schmidt M, Velasco-Salas ZI, Arias L, et al. Health Seeking Behaviour and Treatment Intentions of Dengue and Fever: A Household Survey of Children and Adults in Venezuela. PLoS Negl Trop Dis. 2015;9(12):e0004237.

10.	Khun S, Manderson L. Health seeking and access to care for children with suspected dengue in Cambodia: an ethnographic study. BMC Public Health. 2007;7:262.

11.	Wong LP, AbuBakar S. Health beliefs and practices related to dengue fever: a focus group study. PLoS Negl Trop Dis. 2013;7(7):e2310.

12.	Thorne S, Kirkham SR, MacDonald-Emes J. Interpretive description: a noncategorical qualitative alternative for developing nursing knowledge. Res Nurs Health. 1997;20(2):169-77.

13.	Glanz K LF, Rimer BK. Health Behavior and Health Education: Theory, Research, and Practice. 4th ed ed. San Francisco: Jossey-Bass; 2008.

14.	Ajzen I. The theory of planned behavior. Organizational Behavior and Human Decision Processes. 1991;50(2):179-211.

15.	Rajapakse S, de Silva NL, Weeratunga P, Rodrigo C, Sigera C, Fernando SD. Carica papaya extract in dengue: a systematic review and meta-analysis. BMC Complement Altern Med. 2019;19(1):265.

16.	Ching S, Ramachandran V, Gew LT, Lim SMS, Sulaiman WAW, Foo YL, et al. Complementary alternative medicine use among patients with dengue fever in the hospital setting: a cross-sectional study in Malaysia. BMC Complement Altern Med. 2016;16:37.

17.	Ismail IS, Hairon SM, Yaacob NM, Besari AM, Abdullah S. Usage of Traditional and Complementary Medicine among Dengue Fever Patients in the Northeast Region of Peninsular Malaysia. Malays J Med Sci. 2019;26(3):90-101.

18.	Liu H, Xu JW, Ai Z, Yu Y, Yu B. Treatment seeking behavior and associated factors of suspected dengue fever among Shan people in eastern Shan special region IV, Myanmar: a cross-sectional study. BMC Health Serv Res. 2020;20(1):318.

19.	Vincent C, Furnham A. Why do patients turn to complementary medicine? An empirical study. Br J Clin Psychol. 1996;35(1):37-48.

20.	Selvarajoo S, Liew JWK, Tan W, Lim XY, Refai WF, Zaki RA, et al. Knowledge, attitude and practice on dengue prevention and dengue seroprevalence in a dengue hotspot in Malaysia: A cross-sectional study. Sci Rep. 2020;10(1):9534.

21.	Sára Z. CZ, Tóth T., Fejes J.B., Pörzse G. . Doctor-Patient Knowledge Transfer: Innovative Technologies and Policy Implications. Journal of Information Engineering and Applications. 2013;3:30-8.

22.	McCarthy DM, Waite KR, Curtis LM, Engel KG, Baker DW, Wolf MS. What did the doctor say? Health literacy and recall of medical instructions. Med Care. 2012;50(4):277-82.

23.	Friedman AJ, Cosby R, Boyko S, Hatton-Bauer J, Turnbull G. Effective teaching strategies and methods of delivery for patient education: a systematic review and practice guideline recommendations. J Cancer Educ. 2011;26(1):12-21.

24.	Atack L, Luke R, Chien E. Evaluation of patient satisfaction with tailored online patient education information. Comput Inform Nurs. 2008;26(5):258-64.

25.	Epstein RM, Street RL, Jr. The values and value of patient-centered care. Ann Fam Med. 2011;9(2):100-3.

26.	Marcus C. Strategies for improving the quality of verbal patient and family education: a review of the literature and creation of the EDUCATE model. Health Psychol Behav Med. 2014;2(1):482-95.

27.	Talevski J, Wong Shee A, Rasmussen B, Kemp G, Beauchamp A. Teach-back: A systematic review of implementation and impacts. PLoS One. 2020;15(4):e0231350.

28.	Ng TC, Teo CH, Toh JY, Dunn AG, Ng CJ, Ang TF, et al. Factors influencing healthcare seeking in patients with dengue: systematic review. Trop Med Int Health. 2021.

29.	Ha JF, Longnecker N. Doctor-patient communication: a review. Ochsner J. 2010;10(1):38-43.

30.	Alyaemni A, Theobald S, Faragher B, Jehan K, Tolhurst R. Gender inequities in health: an exploratory qualitative study of Saudi women's perceptions. Women Health. 2013;53(7):741-59.

31.	Gaur P, Kuo M, Kho KA. Demonstrating Lack of Child Care as a Barrier to Health Care for Women in Parkland Health & Hospital System [04H]. Obstetrics & Gynecology. 2020;135:82S.

32.	Nair SK, Dash U. Unravelling the Contextual Factors Mediating Illness Response Using Mixed Methodology:The Case of Re-emerging Infectious Disease in Kerala, India. Journal of Health Management. 2018;20(2):122-35.

33.	Alvarez KS, Bhavan K, Mathew S, Johnson C, McCarthy A, Garcia B, et al. Addressing childcare as a barrier to healthcare access through community partnerships in a large public health system. BMJ Open Qual. 2022;11(4).

34.	Wharton-Smith A, Green J, Loh EC, Gorrie A, Omar SFS, Bacchus L, et al. Using clinical practice guidelines to manage dengue: a qualitative study in a Malaysian hospital. BMC Infect Dis. 2019;19(1):45.

35.	World Health Organization Regional Office for South-East Asia. Comprehensive Guideline for Prevention and Control of Dengue and Dengue Haemorrhagic Fever2011 April 14, 2022]. Available from: <https://apps.who.int/iris/handle/10665/204894>.

**Supporting information**

S1 – Interview guide for doctors

S2 – Interview guide for patients
