# Supplementary material for: Self-care practices and health-seeking behaviours in patients with dengue fever: A qualitative study from patients’ and physicians’ perspectives
Source: PLoS Negl Trop Dis. 2023 Apr 27;17(4):e0011302. doi: 10.1371/journal.pntd.0011302 (PMC10168551; doi:10.1371/journal.pntd.0011302)
Supplement: Attachment — Submitted filename: Replies to reviewers.docx [file pntd.0011302.s005.docx]

**Replies to reviewers**

**Manuscript number: PNTD-D-22-01181R1**

**(the page and line number mentioned below are based on the clean copy version of the manuscript)**

**Comment:**

Would it be appropriate say "use of traditional remedies" instead of "complementary diet". Related to this for readers in other parts of the world, could you provide some descriptions of what exactly "Papaya leaf extract" is and in what form it is ingested. Is it another drink from boiling papaya leaves, or is it a pill or supplement. Crab soup seems like crab soup. I work in Latin America and there are a host of local remedies that all amount to being liquids.

**Reply:**

We agree that ‘traditional remedies’ is a more suitable and relatable term. Thank you for the suggestion.

We have described how papaya leaf was commonly ingested in our patients:

Our patients consumed papaya leaf both in the form of juice (boiling water together with papaya leaf) and pill extract. Papaya leaf juice was more commonly ingested. (Page 10, line 209-210)

**Comment:**

Honestly, I can't understand why a physician would discourage ingesting liquids (not sure about the papaya extract). I want to scold the physicians but if the papaya extract is a pill then it would clearly be optional. There are two issues here: This patients follow Drs advice about increasing fluid intake or not and if they decided to do it with traditionally recognized remedies. It is important to distinguish which involved ingesting liquids and which did not. How were the doctors making this distinction.

**Reply:**

Generally the patients in our study adhered to their doctor’s advice to increase fluid intake but took additional remedies to complement the fluid intake. We added a statement to explain this:

Our patients generally still heeded the advice from their doctors to increase their fluid intake, but took traditional remedies to complement the fluid. (page 11, line 212-214)

We added a quote by Doctor 3 to explain that doctor in our study made a distinction between fluid-based remedies versus the traditional medicine in pill form:

*“If they can’t drink water, drink whatever fluid that is available, as long as juice, juice also is okay. As long as it is fluid, but definitely not traditional medicine.” (Doctor 3, female, 10 years of experience)* (page 13, line 264-266)

We also highlighted a quote by Doctor 11 to explain her concern and why she did not encourage traditional remedies:

Doctor 11 pointed out that she did not encourage intake of traditional remedies. She did not want to give a wrong impression to her patients that the traditional remedies were the substitute for the usual self-care for dengue such as self-monitoring of warning signs and adequate fluid intake. (page 13, line 272-275)

*“So I don’t actually encourage, but if they want to take it, I don’t do anything. Basically I don’t encourage. I don’t want to give the point for them to just take it (traditional remedy) without taking care of other factors that are more important like the warning signs, the fluid intake everything.” (Doctor 11, female, 9 years of experience)* (page 13, line 276-279)

We also discussed about the concern of misconception among the patients for traditional remedies as potential cure for dengue in the Result and Discussion sections:

She also shared an anecdote she heard from others that papaya leaf could help to boost immunity in dengue. (page 11, line 216-217)

*"They also made like, you know, moms being moms, they make weird type of soup for you to drink, like crab soup I don't know what the hell is that? So basically that and I also, I guess they also heard from other people that, you know, if you are having dengue, you have very weak immune system, you should be taking things like papaya extract pill." (Patient 3, 25-year-old female, not admitted)* (page 11, line 218-222)

We have updated our discussion on the traditional remedies:

The discouragement may be due to the doctors’ concern that their patients may focus on traditional remedies and disregard the usual self-care for dengue, which constitutes measures such as adequate fluid intake and self-monitoring of warning signs, as pointed out by Doctor 11. This may create tension in the consultation, as pointed out by some doctors and patients in our study. Perhaps, a more appropriate approach would be to take the ‘middle ground’ stand, as practised by some of our doctors in this study, where doctors can acknowledge the ‘no harm’ perception by patients but also inform them regarding the absence of benefit. The patients can then decide for themselves on traditional remedies. This of course applies only to dietary practices which do not show harm, as pointed out by some doctors in our study who did not stop their patients from taking traditional dietary remedies in the fluid form. The doctors’ concern were more of the traditional remedies in the form of medicine or pill. Addressing traditional remediess and practices in consultation, such as the papaya leaf extract, is important owing to their popularity in countries endemic for dengue such as India, Indonesia and Malaysia (15). While there were some evidence to show that papaya leaf extract improved platelet count, it may not translate to clinical improvement (15, 16). Such misconception may influence people to treat themselves at home without seeing the doctor. (page 20-21, line 446-461)
