# Supplementary material for: Self-care practices and health-seeking behaviours in patients with dengue fever: A qualitative study from patients’ and physicians’ perspectives
Source: PLoS Negl Trop Dis. 2023 Apr 27;17(4):e0011302. doi: 10.1371/journal.pntd.0011302 (PMC10168551; doi:10.1371/journal.pntd.0011302)
Supplement: Attachment — Submitted filename: Replies to editor.docx [file pntd.0011302.s007.docx]

**Replies to reviewers**

**Manuscript number: PNTD-D-22-01181R2**

**(the page and line number mentioned below are based on the clean copy version of the manuscript)**

Dear editors,

Thank you very much for the editing work that you have done for our manuscript.

Overall we are happy with the changes made and we only made some minor rephrasing of the sentences. The following are the replies to some of the specific comments in the manuscript:

1. Suggestion for the following sentence in the Results section for Theme 1: Use of traditional remedies for dengue – “Neither patients or physicians clearly acknowledged that many traditional remedies came in the form of fluids representing a legitimate way to increase fluid intake.”

**Reply:** We discussed this among the research team and decided to omit this as we felt that we didn't really explore it this way in our interviews. It would have been interesting if so, thank you for the suggestion.

2. Clarification on the patient’s quote in Theme 1, whether the patient was distinguishing papaya leaf extract from juice: Use of traditional remedies for dengue:

*"The doctor asked me to drink, to not to drink papaya leaf juice., the extract, yes. She told me not to do that, because it was very common among the patients. But my parents ended up giving it to me, but I did, I said I didn't want to, but I still drink it anyway." (Patient 5, 20-year-old male, admitted)*

**Reply**: What the patient actually meant was the doctor only asked her to drink plain water and avoid drinking papaya leaf juice. She used the word 'juice' and 'extract' in similar context to imply fluid derived from boiling papaya leaf. We edited the quote slightly to avoid confusion:

*"The doctor asked me to drink (plain water), not to drink papaya leaf juice. She told me not to do that, because it was very common among the patients. But my parents ended up giving it to me, but I did, I said I didn't want to, but I still drink it anyway." (Patient 5, 20-year-old male, admitted)*(Page 11, line 232-235)

3. Suggestion to reduce the discussion section to avoid detraction from the key messages

**Reply:** We agree with the removal of the segment highlighted in green. Those points highlighted in green did not emerge directly from our study findings and we do not have to dwell on them in detail.

4. We did some minor rephrasing of the following sentences in Discussion and Conclusion:

| **Original sentence** | **Rephrased sentence** |
| --- | --- |
| Perhaps, the more appropriate approach, expressed by some of our physician participants would be to take the ‘middle ground’ stand, where doctors can acknowledge that these remedies cause ‘no harm’ but do not provide additional benefit beyond the need to ingest liquids. Although, these remedies do not have specific physiological benefits, for example they do not affect the immune response, patients should be encouraged to decide for themselves on the traditional remedies if they also ensure adequate fluid intake and monitor themselves for warning signs. | A plausible clinical approach to the use of traditional remedies, as expressed by some of our physician participants, would be to take the ‘middle ground’ stand, where physicians can acknowledge that these remedies cause ‘no harm’ but do not provide additional benefit beyond the need to ingest liquids. Although these remedies do not have specific physiological benefits, for example they do not enhance the immune response, patients can be counselled to decide for themselves on the traditional remedies, with emphasis on ensuring adequate fluid intake and monitoring themselves for warning signs. **(page 21, line 455-461)** |
| Secondly, explaining to patients that the symptoms they experience because of their immune system is functioning not because of a “weak” immune system to discourage remedies in the form of pills. | Secondly, it is important to explain to patients that the symptoms they experience are because their immune system is functioning, not because of a “weak” immune system. This will discourage the intention to use remedies in the form of pills to boost immune system. **(page 21, line 463-466)** |
| Additionally, physicians need to understand the desperation that influences patients to resort to alternative treatment. Because treatments for dengue are not curative but focus on alleviating symptoms (fever reducers and pain relievers) and maintaining fluid levels symptoms often persist leading to perceived ineffectiveness of conventional treatments driving patients to look for alternative treatments. | Additionally, physicians need to understand the factors that influence patients to consider alternative treatment. The treatment for dengue is not curative but mainly supportive (alleviation of symptoms and fluid therapy). Patients may perceive that the modern treatment for dengue is ineffective and not definitive, driving them to look for alternative treatments. **(page 22, line 478-481)** |
| All the participating physicians emphasized information on warning signs to their patients, but it was clear that this information was not retained with some patients unable to recall any warning signs provided by their physicians. | All the participating physicians emphasized information on warning signs to their patients and provided similar information in a written note for patients to take home. However, it was clear that this information was not retained with some patients unable to recall any warning signs provided by their physicians. **(page 22-23, line 498-501)** |
| The concept of remote monitoring for dengue such as virtual consultation or mobile application for self-monitoring is interesting. However, the safety and efficacy of such intervention should be vigorously studied first before implementation. | More research is needed to explore newer ways to manage outpatient dengue better. The concept of remote monitoring for dengue such as virtual consultation or mobile application for self-monitoring is interesting, with more data needed on its safety and efficacy. **(page 25, line 556-558)** |
| Our findings are applicable beyond Malaysia and illustrate the need for find alternatives to monitoring patients daily through hospital visits and although the importance of emphasizing warning signs to patients is widely promoted worldwide assessment of messaging is needed. | Our findings are applicable beyond Malaysia and illustrate the need for alternatives to monitoring patients daily through hospital or clinic visits. While the importance of promoting awareness of dengue warning signs is recognized worldwide, there is a need to assess whether the message is communicated effectively to different populations. **(page 25, line 564-568)** |
| Conclusion:  There are gaps in patients’ knowledge of dengue and perceptions around self-care practices, health-seeking behaviour and outpatient management of dengue often differed between doctors and patients. Addressing these gaps in patient knowledge and distinct perceptions are needed to improve the safety and delivery of outpatient care for dengue patients. | Conclusion:  Perceptions around self-care practices, health-seeking behavior and outpatient management of dengue often differed between physicians and patients, especially in the comprehension of information about dengue warning signs. We need to rethink our health communication strategies on the traditional remedies, dengue warning signs and timely health-seeking in dengue. Addressing these gaps between patient and physician perceptions and recognition of patient drivers of health-seeking behavior are needed to improve the safety and delivery of outpatient care for dengue patients. **(page 26, line 574-580)** |

5. We replaced the term ‘doctor’ with ‘physician’ to standardize the usage throughout the manuscript. We only retained the term ‘Doctor’ for the participants’ pseudonyms (e.g. Doctor 1, Doctor 2, Doctor 3……).
6. We also rechecked our references and amended the reference style to square bracket as opposed to parentheses previously.
